# Issue Information

**DOI:** 10.1111/jcmm.12141

**Published:** 2014-07-30

**Authors:** LM Popescu

**Affiliations:** Head, Department of Cellular and Molecular Medicine, Faculty of Medicine ‘Carol Davila’ University of Medicine and PharmacyBucharest, Romania Tel: +40 744 535 298; Fax: +40 21 312 4885; E-mail: LMP@jcmm.org

## Editorial Board

**P. Anversa**, Boston, USA

**J.J.M. Bergeron**, Montreal, Canada

**M. Bhatia**, Christchurch, New Zealand

**O. Binah**, Haifa, Israel

**H.E. Blum**, Freiburg, Germany

**T.B. Bolton**, London, UK

**C. Bona**, New York, USA

**M.D. Bootman**, Cambridge, UK

**N. Bornstein**, Tel Aviv, Israel

**G. Bussolati**, Turin, Italy

**K. Camphausen**, Bethesda, USA

**M. Caraglia**, Naples, Italy

**Y.H. Chen**, Shanghai, China

**C.-M. Cheng**, Hsinchu, Taiwan

**S.N. Constantinescu**, Brussels, Belgium

**P. Doevendans**, Utrecht, The Netherlands

**B. Eyden**, Manchester, UK

**M.S. Faussone-Pellegrini**, Florence, Italy

**W. Franke**, Heidelberg, Germany

**L. Frati**, Rome, Italy

**T. Fujimoto**, Nagoya, Japan

**P.M. Glazer**, New Haven, USA

**J. Gooch**, Atlanta, USA

**H. zur Hausen**, Heidelberg, Germany

**A.M. Hofer**, Boston, USA

**R.E. Horch**, Erlangen, Germany

**O. Hovatta**, Huddinge, Sweden

**S.S. Hu**, Beijing, China

**J.D. Huizinga**, Hamilton, Canada

**M. Ivan**, Indianapolis, USA

**B. Jena**, Detroit, USA

**Y.T. Konttinen**, Helsinki, Finland

**T. Kornberg**, San Francisco, USA

**S. Kostin**, Bad Nauheim, Germany

**R.C. Kukreja**, Richmond, VA, USA

**R. Langer**, Cambridge, USA

**J.W. Lawler**, Boston, USA

**F. Lupu**, Oklahoma City, USA

**J. Meldolesi**, Milan, Italy

**M. Mercola**, San Diego, USA

**V.M. Miller**, Rochester, USA

**K. Morgan**, Boston, USA

**J.F. Morris**, Oxford, UK

**C. Mummery**, Utrecht, The Netherlands

**F. Murad**, Houston, USA

**D.F. Mureşanu**, Cluj-Napoca, Romania

**H. O’Neil**, Canberra, Australia

**K.P. Nephew**, Bloomington, USA

**M. Pesce**, Milan, Italy

**O.H. Petersen**, Liverpool, UK

**N.C. Popescu**, Bethesda, USA

**D. Pozo**, Sevilla, Spain

**M.Z. Ratajczak**, Louisville, USA

**U. Ripamonti**, Johannesburg, South Africa

**J. Rubin**, Bethesda, USA

**A. Samali**, Galway, Ireland

**P.R. Sanberg**, Tampa, USA

**R.C. dos Santos Goldenberg** Rio de Janeiro, Brasil

**M. Simionescu**, Bucharest, Romania

**G.W. Sledge**, Indiana, USA

**R.V. Stan**, Hanover, USA

**G. Steinhoff**, Rostock, Germany

**M. Stürzl**, Erlangen, Germany

**M. Taggart**, Newcastle upon Tyne, UK

**A. Tosaki**, Debrecen, Hungary

**N.A. Turner**, Leeds, UK

**C.A. Vacanti**, Boston, USA

**A. Vaheri**, Helsinki, Finland

L. Vécsei, Szeged, Hungary

**V. Velculescu**, Baltimore, USA

**C. Wang**, Beijing, China

**R.A. Wang**, Xi’an, China

**B. Winblad**, Stockholm, Sweden

**C. Zhang**, Newark, USA

## Executive Assistant Editor:

**M. Leabu**, Bucharest, Romania

## Executive Assistant Editor:

Asia Office

**X. Wang**, Shanghai, China

## Assistant Editors:

**H. Amin**, Houston, USA

**K. Cheng**, Los Angeles, CA, USA

**L. Chirieac**, Boston, USA

**Sanda M. Ciontea**, Bucharest, Romania

**V. Cismasiu**, Oxford, UK

**Camelia Iancu-Rubin**, New York, USA

**Elisa-Anamaria Liehn**, Aachen, Germany

**N.I. Moldovan**, Columbus, USA

**M. Oprica**, Stockholm, Sweden

**A.G. Papavassiliou**, Athens, Greece

**R.A. Towner**, Oklahoma City, USA

## Publishing Editors:

**D. Cretoiu**, Bucharest, Romania

**C.G. Manole**, Bucharest, Romania

## Consultant Editor:

**M.E. Hinescu,** Bucharest, Romania

## Editorial Office

*Journal of Cellular and Molecular Medicine*, P.O. Box 35-29, Bucharest 35, Romania. Tel./Fax: +40 21 312 4885. E-mail: editors@jcmm.org

## Publisher

*Journal of Cellular and Molecular Medicine*, Blackwell Publishing Ltd, 9600 Garsington Road, Oxford, OX4 2DQ UK. Tel.: +44 1865 776868 Fax: +44 1865 714591.

Production Editor: Jeremy Chua (jcmm@wiley.com)

## A Journal of Translational Medicine

The *Journal of Cellular and Molecular Medicine* is an international journal publishing peer-reviewed articles dedicated to original research and concepts in all fields of cellular and molecular medicine. Bridging physiology and cellular medicine, and molecular biology and molecular therapeutics, *Journal of Cellular and Molecular Medicine* publishes basic research that furthers our understanding of the cellular and molecular mechanisms of disease and translational studies that convert this knowledge into therapeutic approaches. The journal welcomes Original Articles, Reviews (which may be in Review Series), Short Communications, Editorials and Points of View, as well as Letters to the Editor. From time to time the journal publishes Retro-views of significant ideas and discoveries, and Commentaries.

## Open Access and Copyright

From12th September 2012 all articles accepted by the Journal of Cellular and Molecular Medicine are fully open access: immediately freely available to read, download and share. All articles accepted from 12th September 2012 are published under the terms of the Creative Commons Attribution License. Articles accepted before this date were published under the agreement as stated in the final article. The Creative Commons Attribution License permits use, distribution and reproduction in any medium, provided the original work is properly cited and allows the commercial use of published articles. Copyright on any research article in a journal published by the Journal of Cellular and Molecular Medicine is retained by the author(s). Authors grant Wiley a license to publish the article and identify itself as the original publisher. Authors also grant any third party the right to use the article freely as long as its integrity is maintained and its original authors, citation details and publisher are identified. Further information about open access license and copyright can be found at http://www.wileyopenaccess.com/details/content/12f25db4c87/Copyright--License.html.

## Purchasing Print Reprints

Print reprints of Wiley Open Access articles can be purchased from corporatesales@wiley.com.

## Disclaimer

The Publisher and Editors cannot be held responsible for errors or any consequences arising from the use of information contained in this journal; the views and opinions expressed do not necessarily reflect those of the Publisher and Editors, neither does the publication of advertisements constitute any endorsements by the Publisher and Editors of the products advertised.

Wiley Open Access articles posted to repositories or websites are without warranty from Wiley of any kind, either express or implied, including, but not limited to, warranties of merchantability, fitness for a particular purpose, or non-infringement. To the fullest extent permitted by law Wiley disclaims all liability for any loss or damage arising out of, or in connection with, the use of or inability to use the content.

## Wiley's Corporate Citizenship initiative statement

Wiley's Corporate Citizenship initiative seeks to address the environmental, social, economic, and ethical challenges faced in our business and which are important to our diverse stakeholder groups. Since launching the initiative, we have focused on sharing our content with those in need, enhancing community philanthropy, reducing our carbon impact, creating global guidelines and best practices for paper use, establishing a vendor code of ethics, and engaging our colleagues and other stakeholders in our efforts.

Follow our progress at http://www.wiley.com/go/citizenship

Journal of Cellular and Molecular Medicine is published in 12 issues per year in an online-only format available at Wiley Online Library. Visit wileyonlinelibrary.com to search the articles and register for table of contents e-mail alerts.

Access to this journal is available free online within institutions in the developing world through the HINARI initiatives with the WHO.For information, visit http://www.healthinternetwork.org

This journal is indexed by Science Citation Index Expanded, Current Contents/Life Sciences, Journal Citation Reports/Science Edition, Biological Abstracts, BIOSIS Previews.

ISSN (1582-4934) (Online)

For submission instructions, subscription and all other information visit: http://onlinelibrary.wiley.com/journal/10.1111/(ISSN)1582-4934

